# Adverse pregnancy outcomes in HIV positive women. A study from a District General Hospital in the UK

**DOI:** 10.1186/1742-4690-9-S1-P138

**Published:** 2012-05-25

**Authors:** Priya Thayaparan, Mohanarathi Kawsar, Thambiah Balachandran

**Affiliations:** 1 Luton and Dunstable Hospital, Luton, UK

## Background

Increasing number of women with HIV are choosing to become pregnant as there is reduction in vertical transmission. However, management of HIV in pregnancy still poses a variety of challenges and adverse pregnancy outcomes are still common. We aimed to explore the factors associated with adverse outcomes of pregnancy in our HIV cohort.

## Methods

It is a retrospective case notes review of all the women attended to our unit and had the HIV care from 2008-2011. A total of 87 women were followed up. Three women had two pregnancies during the study period. Data collected from Genitourinary Medicine and maternity records were analysed by using SPSS program.

## Results

Mean age was 34 yrs ranging from 20-43 yrs. Majority (91%) were of African origin; 67% had HIV subtype C; 26% resistant to one or more class of HIV drugs; 55% had a nadir CD4 fewer than 350; 44% diagnosed at an antenatal setting and 62% were planned pregnancies. Prior to the current pregnancy, these women had 121 children: 5% of the children have HIV and 33% not tested for HIV.

None of the children born during the study period were infected with HIV; there were 3 sets of twins; one still birth and one child died soon after birth.

Around 46% were on anti retroviral therapy (ART) during conception, 6% had miscarriage and 16% had emergency caesarean section.

38% of the women experienced an obstetric complication, premature labour 9%; premature rupture of membranes and gestational diabetes both accounted to 4% whilst 3% had post partum haemorrhage.

On ART during conception and late HIV diagnosis, nadir CD4, less than 350 cells were significantly associated (P< 0.05) with having a foetal complication such as prematurity 8%, low birth weight 7% or having a foetal abnormality 2.3%. More analysis is awaited as to drug exposure and adverse outcomes.

## Conclusion

Late diagnosis of HIV and ART during conception is significantly associated with adverse outcomes of pregnancy. Widespread HIV testing is essential and has to be extended to non traditional settings.

